# Supplementary material for: Women’s knowledge, attitudes and views of preconception health and intervention delivery methods: a cross-sectional survey
Source: BMC Pregnancy Childbirth. 2022 Sep 24;22:729. doi: 10.1186/s12884-022-05058-3 (PMC9508727; doi:10.1186/s12884-022-05058-3)
Supplement: Supplementary file 1 — Additional file 1. Questions stems, response options, sources, psychometric properties, and modifications made to the survey’s questionnaire items. [file 12884_2022_5058_MOESM1_ESM.docx]

**Additional file 1: Questions stems, response options, sources, psychometric properties, and modifications made to the survey’s questionnaire items**

1. ***Exposure variables***

| **Variable** | **Question stem and response options** | **Item source, existing psychometric properties, and modifications made** |
| --- | --- | --- |
| Age | *What is your age?*  18-19, 20-24, 25-29, 30-34, 35-39, 40-44, or 45-48 years | Determined using the Government Statistical Service’s Age Band Group 1 D harmonised principle groupings ([1](#_ENREF_1)), with the final option modified in line with the survey’s upper age limit. |
| Educational attainment | *What is the highest level of education you have completed?*  University, Intermediate between secondary school and university (e.g. technical training), Secondary school, Primary school (or less), Still in education /not yet finished | Determined using an adaption of the ‘*what is the highest level of education you have completed?*’ item used by the World Health Organisation’s MONICA project ([2](#_ENREF_2)), with the addition of the response option ‘*still in education/not yet finished’*. No validity or reliability properties were reported for the questionnaire used by the MONICA project ([2](#_ENREF_2)). |
| Ethnicity | *What is your ethnic group? Circle ONE option that best describes your ethnic group or background*  White, Mixed/Multiple ethnic groups, Asian/Asian British, Black/African/Caribbean/Black British, Other ethnic group, please describe | Determined using the Government Statistical Service’s recommended ethnic group question for use in surveys in England ([1](#_ENREF_1)). Only the higher-level ethnicity groupings were used as response options, but with ‘*other ethnic group*’ replaced with ‘*other ethnic group, please describe:..*’ to allow participants who were unsure of their ethnicity grouping to be categorised by the study team. This question arose from a two-year cross-government stakeholder consultation programme ([3](#_ENREF_3)). |
| Country of birth | *What is your country of birth?*  The UK, Other (please write) | Determined using the Government Statistical Service’s harmonised principle items for ‘*country of birth*’ and ‘*year and of arrival in the UK*’ ([1](#_ENREF_1)). |
| Household income | *What was your total household income last year? (before taxes or deductions)*  Less than £13,000, £13,000-£18,999, £19,000-£25,999, £26,000-£31,999, £32,000-£47,999, £48,000-£63,999, £64,000-£95,999, More than £96,000 | Determined using a self-developed item (‘*what was your total household income last year? (before taxes or deductions)*’), with response options derived from the Office of National Statistics’ ‘*Gross household income, UK, financial year ending 2018*' report ([4](#_ENREF_4)), to allow a comparison between the survey respondents and the wider UK population. |

| **Variable** | | **Question stem and response options** | **Item source, existing psychometric properties, and modifications made** | |
| --- | --- | --- | --- | --- |
| Gravidity | | *Have you ever been pregnant before? (circle ONE option)*  Yes, I am pregnant now and this is my first pregnancy, No | Determined using an adaption of the ‘*Everpreg*’ item in the third British national survey of sexual attitudes and lifestyle (Natsal-3) questionnaire ([5](#_ENREF_5)). The response option ‘*I am pregnant now and this is my first pregnancy*’ was added to identify participants who had experience of becoming pregnant but would not be expected to report pregnancy outcomes for the proceeding item. Participants who selected ‘*no*’ were classed as nulligravida. The Natsal-3 questionnaire has been validated for use in UK populations through cognitive interviewing and two large pilot studies ([5](#_ENREF_5)). | |
| Previous Live Birth(s)  Adverse Pregnancy Outcomes | | *How many of each of the following have you had? (enter ‘0’ for anything you have not experienced)*  Miscarriage (or an ectopic pregnancy), Termination or abortion due to foetal abnormalities, Termination or abortion for other reasons, Stillbirth (a baby born after 24 weeks of pregnancy that died before birth), A live birth/baby | Determined using an adaption of the Natsal-3 questionnaire’s ‘PregOL’ item ([5](#_ENREF_5)). ‘*What was the outcome of that pregnancy?*’ was replaced with ‘*How many of each of the following have you had? (enter ‘0’ for anything you have not experienced)*’ as the question stem. The ‘*live birth (one child)*’ and ‘*live birth (more than one child)*’ response options were collapsed to form a singular ‘*live birth(s)*’ option, and the ‘*termination or an abortion*’ response option was split into separate ‘…*due to foetal abnormalities*’ and ‘…*for other reason(s)*’ options. Participants who reported one or more miscarriages, stillbirths and/or terminations due to foetal abnormalities were categorised as having experienced an adverse pregnancy outcome. | |
| Previous infertility | | *Circle ONE number for each of the following two questions: Have you ever had a time, lasting 12 months or longer, when you and a partner were trying for a pregnancy but it didn't happen? Have you or a partner ever sought medical or professional help about infertility?*  Yes, No | Determined using the Natsal-3 questionnaire’s ‘*Infrt1y*’ and ‘*MedHelp*’ items ([5](#_ENREF_5)). Participants who respond ‘*yes*’ to either of these items were categorised as having experienced fertility issues | |
| **Variable** | **Question stem and response options** | | | **Item source, existing psychometric properties, and modifications made** |
| Pregnancy intentions | *Which of these statements best describes the way you feel about having (more) children? (Circle ONE number)*  I would definitely like (more) children, and I’m currently trying,  I would definitely like (more) children, I’m not currently trying, but I would like to get pregnant in the next 1-2 years  I would definitely like (more) children. I’m not currently trying, but I would like to get pregnant in the next 3+years,  I might like (more) children in the future, I’m not sure yet I would definitely not like (more) children,  Don’t know, I’m not able to get pregnant | | | Determined using an adaption of Natsal-3’s ‘*FertInt*’ item ([5](#_ENREF_5)). The response option ‘*I would definitely like (more) children, but I’m not currently trying*’ was split to form separate ‘*I would definitely like (more) children. I’m not currently trying, but I would like to get pregnant in the next 1-2 years*’ and ‘…*in the next 3+ years*’ options, to better capture the immediacy of participants’ pregnancy desire. The option ‘*I’m not able to get pregnant*’ was also added following validity testing. |

1. ***Outcome variables***

| **Variable** | **Question stem and response options** | **Item source, existing psychometric properties, and modifications made** |
| --- | --- | --- |
| Knowledge of preconception risk factors | *List up to 5 things you think a woman can DO (or START or CONTINUE doing) BEFORE pregnancy to help her to have a healthy pregnancy and healthy baby*  *List up to 5 things you think a woman can STOP doing (or AVOID) BEFORE pregnancy to help her to have a healthy pregnancy and healthy baby*  *List ANY OTHER things BEFORE pregnancy that might affect whether a woman has a healthy pregnancy and baby. These might be about her health, her life or her circumstances before pregnancy, or other things.* | Only one identified study – Stern et al. ([6](#_ENREF_6)) - assessed knowledge of preconception risk factors using an open-ended, free-text question. The authors asked only about individual-level lifestyle changes and assigned ‘points’ for eight risk factors. The remaining studies asked specific questions about specific preconception exposures (e.g. “*which of the following actions have you heard are the most important things for women to do before they get pregnant?... Avoid smoking cigarettes”* ([7](#_ENREF_7))), bestowing salience to these factors and likely resulting in an overestimation of knowledge by eliciting desired responses.  The item from Stern et al. ([6](#_ENREF_6)), piloted for use in a population of reproductive-age Swedish women and reviewed by academics, clinicians and laypeople, was therefore modified to form multiple items asking participants to list things that, before pregnancy, can be: (1) done, started or continued; (2) stopped or avoided; or that (3) relate to a woman’s life, circumstances or health, which might affect pregnancy outcomes.  For each exposure identified in a recent umbrella review of preconception exposures ([8](#_ENREF_8)), for which high, moderate or low certainty evidence of a preconceptional association with an adverse pregnancy, birth and postpartum outcome(s) was found, participants were scored as ‘yes’ or ‘no’ depending on whether they had listed it. |
| Perceived awareness of preconception health behaviours | *How aware are you of the positive behaviours and other actions women can take BEFORE pregnancy to help to have a healthy pregnancy and a healthy baby? (Circle ONE of the options)*  Very aware, Moderately aware, Slightly aware, Not aware at all | Of the survey studies identified, the item ‘*to what extent do you feel you are aware of the positive behaviours and other actions pregnant women can take to increase their odds of having a healthy pregnancy and a healthy baby?*’ in Delgado et al. ([9](#_ENREF_9)), scored on a four-point Likert scale, was deemed the most suitable for this variable. The beginning of the question stem was modified to ‘*How aware are you of…can take BEFORE pregnancy to help to have a…*’ to make the wording less complex, less leading, and more specific to the preconception period.  No validity or reliability properties were reported for the questionnaire used in Delgado et al. ([9](#_ENREF_9)) |

| **Variable** | **Question stem and response options** | **Item source, existing psychometric properties, and modifications made** |
| --- | --- | --- |
| Perceived importance of preconception health | *Please rate how much you agree with the following (circle ONE option): “A woman’s health BEFORE pregnancy can affect the health of her and her baby during and after pregnancy”*  Strongly agree, Agree, Neither agree nor disagree, Disagree, Strongly disagree | Of the survey studies identified, the item ‘*A woman’s health before conception can have serious consequences to the health of the baby*’ in Best Start Resource Center’s questionnaire ([10](#_ENREF_10)) was deemed the most suitable for this variable. Its question stem was modified to ‘*A woman’s health BEFORE pregnancy can affect the health of her and her baby during and after pregnancy*’ to be more lay and applicable to both child and maternal pregnancy outcomes.  No validity or reliability properties were reported for the questionnaire used by Best Start Resource Center ([10](#_ENREF_10)). |
| Interest in preconception health education | *How interested are you in knowing more about pre-pregnancy health? (Circle ONE option)*  Very interested, Moderately interested, Slightly interested, Not at all interested | Of the survey studies identified, the item ‘*Are you interested in receiving preconception health education?*’ in Frey et al. ([11](#_ENREF_11)) was deemed the most suitable for this variable. Its question stem was modified to ‘*How interested are you in knowing more about pre-pregnancy health?*’ to be less leading and complex, and the response options were changed to ‘*Very*’, ‘*Moderately*’, ‘*Slightly*’ and ‘*Not at all*’ interested, in line with the response options for ‘*Perceived Importance of Preconception Health’*. |
| Preconcept-ional self-efficacy | *Imagine that you were planning to become pregnant within 6 months. How much do you agree with the following statement? (circle ONE option): “There are things I can do before I become pregnant to help make sure my child is born healthy”*  Strongly agree, Agree, Neither agree nor disagree, Disagree, Strongly disagree | Determined using the Preconceptional Control item in Weisman et al. ([12](#_ENREF_12)). The item introduction (‘*Imagine that you were planning to become pregnant within 6 months*’) was derived from the ‘*Pregnancy planning as cue to action*’ item in Stern et al. ([6](#_ENREF_6)). Scores on this item have been positively associated with folic acid use and receipt of pregnancy planning counselling ([12](#_ENREF_12)). Dose-response effects were also reported for this item, for each additional session attended of a behaviour change intervention to improve the preconceptional and inter-conceptional health of women (odds ratio [OR] 1.31, *p*=0.002), alongside significant dose-response effects for several health behaviours such as checking food labels for nutritional information (OR 1.16, *p*=0.015), partaking in relaxation exercise or meditation for stress management (OR 1.24, *p*= 0.009), and daily use of a multivitamin with folic acid (OR 1.45, *p*=0.009) ([13](#_ENREF_13)). |

| **Variable** | **Question stem and response options** | **Item source, existing psychometric properties, and modifications made** |
| --- | --- | --- |
| Preconception health intentions | *Imagine that you were planning to become pregnant within 6 months. How likely is it that you would make any lifestyle changes during these 6 months, in preparation for pregnancy? (circle ONE)*  Very likely, Quite likely, Neither likely nor unlikely, Quite unlikely, Very unlikely | Determined using the ‘*Pregnancy planning as cue to action’* item in Stern et al. ([6](#_ENREF_6)), scored on a five-point Likert scale.  The questionnaire used in Stern et al. ([6](#_ENREF_6)) was piloted for use in a population of reproductive-age Swedish women |
| Intervention delivery method acceptability  (People/staff groups) | *Please rate how comfortable you would be with discussing health before pregnancy and your personal pregnancy plans with each of the following people (circle ONE of the following options for each person)*  Very comfortable, Somewhat comfortable, Neither comfortable nor uncomfortable, Somewhat uncomfortable, Very uncomfortable | Determined using a self-developed item asking participants to rate, on a five-point Likert scale, how comfortable they would be with discussing preconception health and their pregnancy intentions with various people. These intervention delivery options were derived from suggestions and interventions identified in the literature as well as discussions within the study team. |
| Intervention delivery method last contact  (People/staff groups) | *Please select when you last spoke with each of the following people (about anything–not just about pregnancy)*  *(circle ONE of the following options for each person)*  Within the last week, Within the last month, Within the last year, 1-3 years ago, 4-5 years ago, More than 5 years ago, Never | Determined using a self-developed item asking participants to select, from seven options, when they last spoke to each of the people listed for the ‘Intervention delivery mode acceptability (People/staff groups)’ item. |
| **Variable** | **Question stem and response options** | **Item source, existing psychometric properties, and modifications made** |
| Intervention delivery method acceptability  (Places/ settings) | *Please rate how acceptable it would be to you if information about health before pregnancy was made available in the following places. Circle ONE letter for each place*  Very acceptable, Somewhat acceptable, Neither acceptable nor unacceptable, Somewhat unacceptable, Very unacceptable | Determined using a self-developed item asking participants to rate, on a five-point Likert scale, how acceptable they felt it would be for information about preconception health to be made available in a number of places or settings.  These intervention delivery options were derived from suggestions and interventions identified in the literature as well as discussions within the study team. |

**References**

1. Government Statistical Service. Harmonised principles by topic. <https://gss.civilservice.gov.uk/guidances/harmonised-standards-guidance/>. Accessed 26 May 2022.

2. World Health Organisation. MONICA Manual, Part III: Population Survey. Section 1: Population Survey Data Component. 1997. <https://www.thl.fi/publications/monica/manual/part3/iii-1.htm>. Accessed 26 May 2022.

3. Office for National Statistics. Ethnic group, national identity and religion. Measuring equality: A guide for the collection and classification of ethnic group, national identity and religion data in the UK. <https://www.ons.gov.uk/methodology/classificationsandstandards/measuringequality/ethnicgroupnationalidentityandreligion>. Accessed 05 May 2022.

4. Office for National Statistics. Gross household income, UK, financial year ending 2018. 2019. <https://www.ons.gov.uk/peoplepopulationandcommunity/personalandhouseholdfinances/incomeandwealth/adhocs/009772grosshouseholdincomeukfinancialyearending2018>. Accessed 26 May 2022.

5. Erens B, Phelps A, Clifton S, Mercer CH, Tanton C, Hussey D, et al. Methodology of the third British national survey of sexual attitudes and lifestyles (Natsal-3). Sex Transm Infect. 2014;90(2):84-9.

6. Stern J, Larsson M, Kristiansson P, Tyden T. Introducing reproductive life plan-based information in contraceptive counselling: an RCT. Hum Reprod. 2013;28(9):2450-61.

7. Mitchell EW, Levis DM, Prue CE. Preconception health: awareness, planning, and communication among a sample of US men and women. Maternal and Child Health Journal. 2012;16(1):31-9.

8. Daly M, Kipping RR, Tinner LE, Sanders J, White JW. Preconception exposures and adverse pregnancy, birth and postpartum outcomes: Umbrella review of systematic reviews. Paediatric and Perinatal Epidemiology. 2021;36(2):288-99.

9. Delgado C. Pregnancy 101: a call for reproductive and prenatal health education in college. Maternal and Child Health Journal. 2013;17(2):240-7.

10. Best Start Resource Center. Preconception Health: Awareness and Behaviours in Ontario. 2009. <https://resources.beststart.org/wp-content/uploads/2018/12/F11-E.pdf>. Accessed 26 April 2022.

11. Frey KA, Files JA. Preconception healthcare: what women know and believe. Maternal and Child Health Journal. 2006;10(1):73-7.

12. Weisman CS, Hillemeier MM, Chase GA, Misra DP, Chuang CH, Parrott R, et al. Women's perceived control of their birth outcomes in the Central Pennsylvania Women's Health Study: implications for the use of preconception care. Women Health Iss. 2008;18(1):17-25.

13. Hillemeier MM, Downs DS, Feinberg ME, Weisman CS, Chuang CH, Parrott R, et al. Improving Women's Preconceptional Health. Findings from a Randomized Trial of the Strong Healthy Women Intervention in the Central Pennsylvania Women's Health Study. Women's Health Issues. 2008;18(6):S87-S96.
