# Supplementary material for: Women’s knowledge, attitudes and views of preconception health and intervention delivery methods: a cross-sectional survey
Source: BMC Pregnancy Childbirth. 2022 Sep 24;22:729. doi: 10.1186/s12884-022-05058-3 (PMC9508727; doi:10.1186/s12884-022-05058-3)
Supplement: Supplementary file 2 — Additional file 2. Intervention delivery methods featured in the study questionnaire, with sources. [file 12884_2022_5058_MOESM2_ESM.docx]

**Additional file 2: Intervention delivery methods featured in the study questionnaire, with sources**

| **Intervention delivery method** | **Source(s)** |
| --- | --- |
| General practitioners (GPs)  Practice nurses  Obstetricians/gynaecologists  Midwives  Health visitors  Pharmacists  Dentists  Community/family support workers  Sexual health/family planning clinic staff  Hairdresser/beauticians  Friend(s)  Family/partners  Television  Billboards  Radio  Printed material in healthcare settings  On social media  By personal text or email (e.g. from a GP)  On preconception health websites/apps  Included with packs of tampons &sanitary pads  Included with pregnancy tests  Included in health education in schools  The workplace | ([1](#_ENREF_1)) ([2](#_ENREF_2)) ([3](#_ENREF_3)) ([4](#_ENREF_4)) ([5](#_ENREF_5))  ([1](#_ENREF_1)) ([2](#_ENREF_2)) ([6](#_ENREF_6)) ([7](#_ENREF_7)) ([8](#_ENREF_8)) ([9](#_ENREF_9))  ([1](#_ENREF_1)) ([4](#_ENREF_4)) ([10](#_ENREF_10)) ([4](#_ENREF_4))  ([1](#_ENREF_1)) ([6](#_ENREF_6)) ([7](#_ENREF_7))  ([7](#_ENREF_7))  ([2](#_ENREF_2)) ([11](#_ENREF_11))  Study team  ([3](#_ENREF_3)) ([11](#_ENREF_11))  ([12](#_ENREF_12)) ([2](#_ENREF_2)) ([13](#_ENREF_13))  ([14](#_ENREF_14))  ([15](#_ENREF_15))  ([11](#_ENREF_11))  ([11](#_ENREF_11)) ([16](#_ENREF_16))  ([14](#_ENREF_14)) ([17](#_ENREF_17))  ([14](#_ENREF_14)) ([17](#_ENREF_17))  ([9](#_ENREF_9))  ([18](#_ENREF_18)) ([11](#_ENREF_11))  ([19](#_ENREF_19))  ([9](#_ENREF_9))  ([3](#_ENREF_3))  ([3](#_ENREF_3))  ([11](#_ENREF_11)) ([3](#_ENREF_3)) ([20](#_ENREF_20)) ([16](#_ENREF_16)) ([15](#_ENREF_15)) ([21](#_ENREF_21)) ([6](#_ENREF_6))  ([16](#_ENREF_16)) ([11](#_ENREF_11)) |

**References**

1. Atrash H, Jack BW, Johnson K, Coonrod DV, Moos M-K, Stubblefield PG, et al. Where is the “W” oman in MCH? American Journal of Obstetrics and Gynecology. 2008;199(6):S259-S65.

2. Shannon GD, Alberg C, Nacul L, Pashayan N. Preconception healthcare delivery at a population level: construction of public health models of preconception care. Maternal and Child Health Journal. 2014;18(6):1512-31.

3. Health Canada. Preconception Care. In: Hanvey L, editors. Family-centered Maternity and Newborn Care: National Guidelines. Ottawa: Minister of Public Works and Government Services; 2000. p. 81-104.

4. Berghella V. Preconception care. In: Berghella V, editors. Obstetric Evidence-Based Guidelines, Second Edition. London: CRC Press; 2012. p. 19-29.

5. French RS, Geary R, Jones K, Glasier A, Mercer CH, Datta J, et al. Where do women and men in Britain obtain contraception? Findings from the third National Survey of Sexual Attitudes and Lifestyles (Natsal-3). BMJ Sex Reprod Health. 2018;44(1):16-26.

6. Misra DP, Grason H. Achieving safe motherhood: applying a life course and multiple determinants perinatal health framework in public health. Women Health Iss. 2006;16(4):159-75.

7. Heyes T, Long S, Mathers N. Preconception care: practice and beliefs of primary care workers. Family Practice. 2004;21(1):22-7.

8. Lynch M, Squiers L, Lewis MA, Moultrie R, Kish-Doto J, Boudewyns V, et al. Understanding women’s preconception health goals: Audience segmentation strategies for a preconception health campaign. Social Marketing Quarterly. 2014;20(3):148-64.

9. Mazza D, Chapman A, Michie S. Barriers to the implementation of preconception care guidelines as perceived by general practitioners: a qualitative study. BMC Health Services Research. 2013;13(1):1-8.

10. Fiore E. March of Dimes updates: is early prenatal care too late. Contemp Ob Gyn. 2002;12:54-72.

11. Government of Canada. Chapter 2: Preconception care. 2017. <https://www.canada.ca/en/public-health/services/publications/healthy-living/maternity-newborn-care-guidelines-chapter-2.html>. Accessed 04 May 2022.

12. Public Health England. Genitourinary Medicine Clinic Activity Dataset (GUMCADv2). 2013. <https://data.gov.uk/dataset/06aa56ce-6686-40e2-a036-c289bb5adbc9/genito-urinary-medicine-clinic-activity-dataset-gumcad>. Accessed 04 May 2022.

13. Paquette R, Tanton C, Burns F, Prah P, Shahmanesh M, Field N, et al. Illicit drug use and its association with key sexual risk behaviours and outcomes: Findings from Britain’s third National Survey of Sexual Attitudes and Lifestyles (Natsal-3). Plos One. 2017;12(5):e0177922.

14. Hussaini KS, Hamm E, Means T. Using community-based participatory mixed methods research to understand preconception health in African American communities of Arizona. Maternal and Child Health Journal. 2013;17(10):1862-71.

15. Brown HK, Mueller M, Edwards S, Mill C, Enders J, Graves L, et al. Preconception health interventions delivered in public health and community settings: A systematic review. Can J Public Health. 2017;108(4):e388-e97.

16. Lassi ZS, Dean SV, Mallick D, Bhutta ZA. Preconception care: delivery strategies and packages for care. Reprod Health. 2014;11(3):1-17.

17. Williams P, McHenery J, McMahon A, Anderson H. Impact evaluation of a folate education campaign with and without the use of a health claim. Australian and New Zealand journal of public health. 2001;25(5):396-404.

18. Mackert M, Kim E, Guadagmo M, Donovan-Kicken E. Using Twitter for prenatal health promotion: encouraging a multivitamin habit among college-aged females. In: Smith AC, editors. Global Telehealth 2012: Delivering Quality Healthcare Anywhere Sydney: IOS Press; 2012. p. 93-103.

19. Milan JE, White AA. Impact of a Stage-Tailored, Web-Based Intervention on Folic Acid—Containing Multivitamin Use by College Women. American Journal of Health Promotion. 2010;24(6):388-95.

20. Boulet SL, Parker C, Atrash H. Preconception care in international settings. Maternal and Child Health Journal. 2006;10(1):29-35.

21. Elsinga J, Van Der Pal-de Bruin KM, Le Cessie S, De Jong-Potjer LC, Verloove-Vanhorick SP, Assendelft WJJ. Preconception counselling initiated by general practitioners in the Netherlands: Reaching couples contemplating pregnancy. BMC Family Practice. 2006;7(1):1-8.
