# Supplementary material for: Women’s knowledge, attitudes and views of preconception health and intervention delivery methods: a cross-sectional survey
Source: BMC Pregnancy Childbirth. 2022 Sep 24;22:729. doi: 10.1186/s12884-022-05058-3 (PMC9508727; doi:10.1186/s12884-022-05058-3)
Supplement: Supplementary file 3 — Additional file 3. Covariates included in each adjusted analysis. [file 12884_2022_5058_MOESM3_ESM.docx]

**Additional file 3: Covariates included in each adjusted analysis**

1. ***Knowledge of preconception risk factors***

| **Outcome** | **Exposure** | **Covariates** |
| --- | --- | --- |
| Knowledge of each preconception risk factor | Age | Household income, Gravidity,  Pregnancy intentions, Infertility |
|  | Household income | Age, Education, Gravidity, Infertility |
|  | Educational attainment | Household income, Gravidity, Pregnancy intentions, Infertility |
|  | Ethnicity | Age, Education, Gravidity |
|  | Country of birth | Age, Education, Gravidity |
|  | Gravidity | Age, Household income, Education, Pregnancy intentions, Infertility |
|  | Livebirth(s) | Age, Household income, Education, Pregnancy intentions, Infertility |
|  | Adverse pregnancy outcomes | Age, Household income, Education, Pregnancy intentions, Infertility |
|  | Infertility | Age, Education, Gravidity |
|  | Pregnancy intentions | Age, Education, Gravidity |

1. ***Attitudes towards preconception health***

| **Outcome** | **Exposure** | **Covariates** |
| --- | --- | --- |
| Perceived awareness of preconception risk factors | Age | Household income, Country of birth, Gravidity, Pregnancy desire, Infertility |
|  | Household income | Age, Gravidity |
|  | Educational attainment | Household income, Country of birth, Gravidity, Pregnancy desire, Infertility |
|  | Ethnicity | Age, Country of birth, Gravidity |
|  | Country of birth | Age, Gravidity, Pregnancy desire |
|  | Gravidity | Age, Household income, Country of birth, Pregnancy desire, Infertility |
|  | Livebirth(s) | Age, Household income, Country of birth, Pregnancy desire, Infertility |
|  | Adverse pregnancy outcomes | Age, Pregnancy desire, Infertility |
|  | Infertility | Age, Gravidity |
|  | Pregnancy intentions | Age, Country of birth, Gravidity |
| Perceived importance of preconception health | Age | Household income, Ethnicity |
|  | Household income | Age |
|  | Educational attainment | Age, Household income |
|  | Ethnicity | Age |
|  | Country of birth | Age, Ethnicity |
|  | Gravidity | Age, Household income, Ethnicity |
|  | Livebirth(s) | Age, Household income |
|  | Adverse pregnancy outcomes | Age |
|  | Infertility | Age |
|  | Pregnancy intentions | Age |
| **Outcome** | **Exposure** | **Covariates** |
| Interest in preconception health education | Age | Household income, Ethnicity, Gravidity, Pregnancy intentions |
|  | Household income | Age, Education, Gravidity |
|  | Educational attainment | Household income, Ethnicity, Gravidity, Pregnancy intentions |
|  | Ethnicity | Household income, Ethnicity, Gravidity, Pregnancy intentions |
|  | Country of birth | Age, Education, Gravidity |
|  | Gravidity | Age, Education, Ethnicity, Household income, Pregnancy intentions |
|  | Livebirth(s) | Age, Education, Household income, Pregnancy intentions |
|  | Adverse pregnancy outcomes | Age, Education, Gravidity, Pregnancy intentions |
|  | Infertility | Age, Education, Gravidity |
|  | Pregnancy intentions | Age, Education, Gravidity |
| Preconception health self-efficacy | Age | Household income, Education, Gravidity |
|  | Household income | Age, Education, Gravidity |
|  | Educational attainment | Household income, Country of birth, Gravidity |
|  | Ethnicity | Age, Education, Country of birth, Gravidity |
|  | Country of birth | Age, Education, Gravidity |
|  | Gravidity | Age, Household income, Education, Country of birth |
|  | Livebirth(s) | Age, Household income, Education, Country of birth |
|  | Adverse pregnancy outcomes | Age, Education |
|  | Infertility | Age, Education, Gravidity |
|  | Pregnancy intentions | Age, Education, Country of birth, Gravidity |
| Preconception lifestyle change intentions | Age | Ethnicity, Country of birth |
|  | Household income | *none* |
|  | Educational attainment | Ethnicity, Country of birth |
|  | Ethnicity | Country birth |
|  | Country of birth | Ethnicity |
|  | Gravidity | Ethnicity, Country of birth |
|  | Livebirth(s) | Country of birth |
|  | Adverse pregnancy outcomes | *none* |
|  | Infertility | *none* |
|  | Pregnancy intentions | Country of birth |
