# Supplementary material for: Women’s knowledge, attitudes and views of preconception health and intervention delivery methods: a cross-sectional survey
Source: BMC Pregnancy Childbirth. 2022 Sep 24;22:729. doi: 10.1186/s12884-022-05058-3 (PMC9508727; doi:10.1186/s12884-022-05058-3)
Supplement: Supplementary file 4 — Additional file 4. ‘Extraneous’ exposures* reported by participants (%), with accepted responses. [file 12884_2022_5058_MOESM4_ESM.docx]

**Additional file 4: ‘Extraneous’ exposures* reported by participants (%), with accepted responses**

| **Preconception exposure** | **%** | **Accepted responses** |
| --- | --- | --- |
| Alcohol | 89.7 | Mentions alcohol or ’drinking’ as something to avoid/reduce/limit |
| Smoking | 89.3 | Mentions smoking, tobacco or second-hand smoke exposure as something to avoid/reduce/limit, or a smoke-free environment as something to aim for |
| Illicit drugs/substance abuse | 48.3 | Mentions (illegal/recreational) drugs or substance abuse as something to avoid/reduce/limit |
| Caffeine | 13.9 | Mentions caffeine, coffee or tea as something to avoid/reduce/limit |
| Avoidance of certain foods | 4.6 | Mentions the avoidance of certain foods (e.g. ‘certain meats’, ‘raw produce’) |
| Dieting | 1.9 | Mentions (crash) dieting or a restrictive diet as something to avoid |
| Hydration | 4.7 | Mentions remaining hydrated or drinking enough water as something to do/start/continue |
| Vitamins | 34.9 | Mentions (multi)vitamins, but does not explicitly mention that these should contain folic acid |
| Vitamin D | 1.8 | Mentions vitamin D as a positive |
| Vitamin B12 | 0.2 | Mentions vitamin B12 as a positive |
| Iron levels | 1.8 | Mentions iron levels or eating foods rich in iron as a positive |
| Omega 3 | 0.2 | Mentions Omega 3 supplementation |
| Hormones | 1.2 | Mentions hormonal (im)balance |
| Physical strain or injury | 10.7 | Mentions strenuous, excessive or extreme exercise, heavy lifting, travelling, ‘overdoing it’, ‘certain exercises’, or other activities involving risk of injury as something to avoid |
| General health and lifestyle | 7.8 | Mentions adopting a healthy ‘lifestyle’, optimising health, remaining healthy, ‘lifestyle changes’, general ‘social life’, ‘partying’, country versus city life, or having ‘unhealthy habits’ |
| Sleep | 16.0 | Mentions regular or sufficient sleep or rest as a positive, or shift work or ‘late nights’ as a negative |
| Stress | 51.3 | Mentions stress (including job stress), catastrophising, trying too hard to get pregnant, stressful life events, or an unstable lifestyle as a negative, or stress management as a positive |
| Self-care/Mental health | 33.1 | Mentions self-care, practising mindfulness, prioritising health or fun, aiming for a ‘balanced lifestyle’, ‘looking after’ oneself, relaxing, enjoying life, or resolving sources of conflict as a positive, or disregarding self-care (e.g. through ‘ignoring stress’) or comparing oneself to others as a negative  Or: Mentions mental health or wellbeing, emotional security or safety, having a ‘good mindset’, speaking to a therapist, or specific mental health considerations or conditions like trauma, post-traumatic stress disorder, anxiety and depression, or eating disorders |
| **Preconception exposure** | **%** | **Accepted responses** |
| Employment circumstances | 10.7 | Mentions job stability, employment terms (e.g. maternity pay), employer support or attitudes, work-life balance, or avoiding long hours or occupational exposure to chemicals or other sources of risk (e.g. cabin crew, manual handling) |
| Social support and relationships | 25.3 | Mentions support from family, friends or colleagues, having people to talk to, joining groups, investing in social relationships, the quality of or issues with these relationships, or avoiding negative people or influence  Or: Mentions the quality or stability of a romantic relationship, having problems with, receiving support from, or discussing concerns with a partner, relationship stress, or continuing to have frequent intercourse or a ‘healthy’ sex life |
| Multiple partners | 0.4 | Mentions having multiple sexual partners as something to avoid |
| Partner’s health | 1.6 | Mentions partner medical history, physical health, or health behaviours |
| Domestic environment | 15.1 | Mentions the home environment (e.g. whether it is clean, dry, safe), housing standards, living situation, environmental instability, or homelessness |
| Pollution | 4.2 | Mentions pollution, toxic substances, harmful chemicals, or fumes as something to avoid |
| Green space | 0.1 | Mentions access to green space |
| Immunisation | 2.2 | Mentions having vaccinations or checking for immunity to viruses |
| Infection risk | 1.1 | Mentions risk of viral infections such as COVID-19 (e.g. through living in an area with high prevalence) as a negative, or taking steps to reduce one’s risk of infection (e.g. through wearing a face covering or avoiding travel to countries with greater infectious disease risk) as a positive |
| Healthcare access and quality | 9.8 | Mentions accessing healthcare, taking advice from or seeing a general practitioner, pharmacist, obstetrician or midwife (e.g. for a check-up, medical tests, or a discussion about preconception health or one’s medical history), an individual’s relationship with these care providers, or the quality of healthcare they receive |
| Health conditions | 21.6 | Mentions physical health conditions or medical issues (e.g. their management), medical history, ‘bad health’, terminal illness or disease, or specific health conditions such as infection, pregestational hypertension, or diabetes |
| Sexual and reproductive health | 6.6 | Mentions sexual health, having a preconception sexual health check, sexually transmitted diseases, cervical screening, menstruation issues, female genital mutilation, or health conditions affecting the reproductive system such as polycystic ovary syndrome or endometriosis |
| Fertility | 1.9 | Mentions fertility issues, becoming informed of potential fertility issues, or having a fertility check |
| **Preconception exposure** | **%** | **Accepted responses** |
| Medications | 12.7 | Mentions prescription or over-the-counter medications, seeing a general practitioner to review current medications, or avoiding self-medication |
| Genetics | 10.3 | Mentions maternal genetics or familial medical history |
| Having/accessing appropriate information | 9.3 | Mentions accessing or seeking appropriate advice or information (e.g. to inform patient choice), having a good knowledge of pregnancy and childbirth and/or associated risks and risk factors, asking others about their experience of pregnancy, or avoiding unsubstantiated claims |
| Contraception | 4.0 | Mentions contraception as something to stop/avoid |
| Oral health | 0.7 | Mentions oral health, looking after one’s teeth, or seeing a dentist |
| Complementary therapy | 0.7 | Mentions complementary therapies such as reflexology, detoxification, or holistic therapy as a positive |
| Socio-economic status | 17.8 | Mentions (socio)economic or financial circumstances, income, being able to afford a pregnancy, or educational attainment |
| Pregnancy intendedness | 0.5 | Mentions pregnancy intendedness or the ‘circumstance of the way [a woman] got pregnant e.g. rape’ |
| Previous pregnancy outcomes | 2.9 | Mentions outcomes or ‘issues’ from previous pregnancies (e.g. miscarriage) |
| Breastfeeding | 0.1 | Mentions breastfeeding as something to stop before becoming pregnant again |
| Pelvic floor exercises | 2.4 | Mentions pelvic floor or Kegel exercises, or ‘maintaining pelvic health’ |
| Menstrual cycle | 2.5 | Mentions monitoring or ‘becoming familiar with’ one’s menstrual/ovulation cycle |
| Preparation for pregnancy/parenthood | 6.7 | Mentions financial (e.g. saving), domestic or mental preparation for pregnancy and associated changes, pregnancy ‘readiness’, ensuring things are ‘in place’, the ability to ‘plan ahead’, considering childcare arrangements, or taking parenting classes |
| Radiation | 0.8 | Mentions sources of radiation such as x-ray machines, mobile phones or microwaves as something to avoid |
| Hygiene | 0.5 | Mentions good hygiene as a positive |

Legend: *Preconception exposures listed by participants for which there was *no* high, moderate or low certainty evidence of an association(s) with an adverse pregnancy, birth or postpartum outcome(s) in Daly et al. ([1](#_ENREF_1))

**References**

1. Daly M, Kipping RR, Tinner LE, Sanders J, White JW. Preconception exposures and adverse pregnancy, birth and postpartum outcomes: Umbrella review of systematic reviews. Paediatric and Perinatal Epidemiology. 2021;36(2):288-99.
