# Supplementary material for: Women’s knowledge, attitudes and views of preconception health and intervention delivery methods: a cross-sectional survey
Source: BMC Pregnancy Childbirth. 2022 Sep 24;22:729. doi: 10.1186/s12884-022-05058-3 (PMC9508727; doi:10.1186/s12884-022-05058-3)
Supplement: Supplementary file 5 — Additional file 5. Proportion of participants who listed each preconception risk factor, by participant characteristic. [file 12884_2022_5058_MOESM5_ESM.docx]

| **Additional file 5: Proportion of participants who listed each preconception risk factor, by participant characteristic** | | | | | | | |
| --- | --- | --- | --- | --- | --- | --- | --- |
| **Characteristic** | **Preconception risk factor** | | | | | | |
|  | **Folic acid** | **Physical activity** | **Body mass index (BMI) / weight** | **Diet -Mediterranean/ high Alternate Healthy Eating Index** | **Abuse*** | **Age** | **Interpregnancy intervals** |
| **Age**  18-24 years  25-29 years  30-34 years  35-39 years  40-48 years | 12.3 (7.5-18.8)  21.5 (16.8-26.8)  42.7 (34.1-51.2)  53.2 (44.6-61.7)  47.2 (38.9-55.7) | 77.4 (69.7-83.9)  75.6 (70.1-80.6)  87.0 (80.0-92.2)  80.6 (73.0-86.8)  79.2 (71.6-85.5) | 28.8 (21.6-36.8)  38.9 (33.1-44.9)  46.6 (37.8-55.5)  42.4 (34.1-51.1)  45.8 (37.5-54.3) | 87.0 (80.4-92.0)  84.7 (79.9-88.8)  88.5 (81.8-93.4)  85.6 (78.7-91.0)  85.4 (78.6-90.7) | 6.8 (3.3-12.2)  3.6 (1.8-6.6)  8.4 (4.3-14.5)  9.4 (5.1-15.5)  6.3 (2.9-11.5) | 4.8 (1.9-9.6)  6.2 (3.6-9.7)  4.6 (1.7-9.7)  7.9 (4.0-13.7)   - 1. (2.4-10.7) | 0.0 (0.0-2.5†)  0.4 (0.0-2.0)  0.8 (0.0-4.2)  0.0 (0.0-2.6)  0.0 (0.0-2.5) |
| **Household income**  <£19,000  £19,000-£25,999  £26,000-£31,999  £32,000-£47,999  £48,000-£63,999  £64,000-£95,999  ≥£96,000 | 25.5 (17.5-34.9)  23.5 (15.0-34.0)  22.5 (13.5-34.0)  30.3 (22.9-38.5)  37.3 (29.8-45.4)  40.4 (32.7-48.4)  41.9 (31.8-52.6) | 69.8 (60.1-78.3)  72.9 (62.2-82.0)  81.7 (70.7-89.9)  79.6 (72.0-85.9)  81.0 (74.0-86.8)  83.2 (76.5-88.6)  81.7 (72.4-89.0) | 31.1 (22.5-40.9)  28.2 (19.0-39.0)  33.8 (23.0-46.0)  37.3 (29.4-45.8)  42.4 (34.6-50.5)  45.3 (37.5-53.3)  57.0 (46.3-67.2) | 82.1 (73.4-88.9)  85.9 (76.6-92.5)  93.0 (84.3-97.7)  85.9 (79.1-91.2)  86.1 (79.7-91.1)  86.3 (80.0-91.2)  83.9 (74.8-90.7) | 7.5 (3.3-14.3)  8.2 (3.4-16.2)  7.0 (2.3-15.7)  4.9 (2.0-9.9)  6.3 (3.1-11.3)  6.2 (3.0-11.1)  5.4 (1.8-12.1) | 5.7 (2.1-11.9)  7.1 (2.6-14.7)  2.8 (0.3-9.8)  4.2 (1.6-9.0)  5.7 (2.6-10.5)  6.8 (3.5-11.9)   - 1. (3.1-14.9) | 0.0 (0.0-3.4)  0.0 (0.0-4.2)  0.0 (0.0-5.1)  0.0 (0.0-2.6)  0.0 (0.0-2.3)  0.6 (0.0-3.4)  1.1 (0.0-5.8) |
| **Ethnicity**  White  Minority ethnicity | 32.9 (29.5-36.3)  34.9 (23.3-48.0) | 79.4 (76.4-82.2)  76.2 (63.8-86.0) | 40.2 (36.7-43.7)  36.5 (24.7-49.6) | 85.9 (83.3-88.3)  87.3 (76.5-94.4) | 6.5 (4.9-8.5)  3.2 (0.4-11.0) | 6.1 (4.5-8.1)  3.2 (0.4-11.0) | 0.3 (0.0-0.9)  0.0 (0.0-5.7) |
| **Education**  School  Intermediate  University | 22.7 (13.3-34.7)  27.0 (20.8-34.0)  36.6 (32.6-40.7) | 78.8 (67.0-87.9)  70.3 (63.1-76.8)  82.6 (79.2-85.6) | 36.4 (24.9-49.1)  41.6 (34.4-49.1)  40.1 (36.0-44.2) | 86.4 (75.7-93.6)  83.8 (77.7-88.8)  87.3 (84.3-90.0) | 10.6 (4.4-20.6)  5.9 (3.0-10.4)  5.8 (4.0-8.0) | 6.1 (1.7-14.8)  5.4 (2.6-9.7)  6.2 (4.3-8.5) | 0.0 (0.0-2.0)  0.4 (0.0-1.3)  0.0 (0.0-21.8) |
| **Country of birth**  United Kingdom  Other | 33.5 (30.1-36.9)  28.4 (18.0-40.7) | 78.8 (75.7-81.6)  86.6 (76.0-93.7) | 40.7 (37.2-44.3)  31.3 (20.6-43.8) | 85.5 (82.8-87.9)  89.6 (79.7-95.7) | 6.7 (5.0-8.7)  3.0 (0.4-10.4) | 5.4 (3.9-7.3)  10.4 (4.3-20.3) | - 1. (0.0-0.7)   1.5 (0.0-8.0) |
| **Ever pregnant**  No  Yes | 19.8 (16.1-23.9)  46.8 (41.8-51.8) | 77.7 (73.4-81.5)  80.7 (76.5-84.4) | 36.7 (32.2-41.5)  43.8 (38.9-48.8) | 85.1 (81.4-88.3)  86.9 (83.2-90.0) | 4.4 (2.7-6.8)  8.4 (5.9-11.6) | 7.0 (4.8-9.8)  4.7 (2.9-7.2) | 0.2 (0.0-1.3)  0.2 (0.0-1.4) |
| **Previous live birth(s)**  No  Yes | 22.4 (18.8-26.2)  49.4 (43.8-55.0) | 78.6 (74.8-82.1)  79.9 (75.2-84.2) | 36.5 (32.3-40.8)  46.0 (40.5-51.6) | 85.3 (81.9-88.3)  87.0 (82.9-90.5) | 4.7 (3.0-6.9)  9.0 (6.1-12.6) | 6.9 (4.8-9.4)  4.3 (2.4-7.1) | 0.2 (0.0-1.1)  0.3 (0.0-1.7) |
| **Characteristic** | **Preconception risk factor** | | | | | | |
|  | **Folic acid** | **Physical activity** | **Body mass index (BMI) / weight** | **Diet -Mediterranean/ high Alternate Healthy Eating Index** | **Abuse** | **Age** | **Interpregnancy intervals** |
| **Adverse outcomes**  No  Yes † | 28.0 (24.6-31.5)  57.1 (48.5-65.5) | 79.7 (76.5-82.6)  76.4 (68.5-83.2) | 39.6 (36.0-43.4)  42.9 (34.5-51.5) | 86.0 (83.2-88.5)  85.7 (78.8-91.1) | 5.9 (4.3-7.9)  8.6 (4.5-14.5) | 6.2 (4.5-8.3)  4.3 (1.6-9.1) | 0.3 (0.0-1.0)  0.0 (0.0-2.6) |
| **Previous infertility**  No  Yes ‡ | 30.8 (27.5-34.4)  47.3 (37.7-57.0) | 79.5 (76.4-82.4)  77.3 (68.3-84.7) | 39.0 (35.4-42.7)  48.2 (38.6-57.9) | 86.0 (83.3-88.5)  86.4 (78.5-92.2) | 6.2 (4.6-8.2)  7.3 (3.2-13.8) | 5.7 (4.1-7.6)  7.3 (3.2-13.8) | 0.3 (0.0-1.0)  0.0 (0.0-3.3) |
| **Pregnancy intentions**  Definitely not  Not sure/Don't know  In the next 3+ years  Within 2 years | 44.3 (37.8-50.9)  27.3 (21.4-33.8)  15.8 (10.9-22.0)  42.8 (35.7-50.1) | 78.3 (72.5-83.4)  78.0 (71.8-83.4)  78.1 (71.4-84.0)  82.0 (75.8-87.1) | 41.7 (35.3-48.3)  38.8 (32.1-45.7)  34.4 (27.6-41.8)  44.8 (37.7-52.1) | 83.8 (78.5-88.3)  90.9 (86.2-94.4)  86.9 (81.1-91.4)  83.5 (77.5-88.4) | 6.4 (3.6-10.3)  10.5 (6.7-15.5)  4.4 (1.9-8.4)  4.1 (1.8-8.0) | 6.4 (3.6-10.3)  6.2 (3.4-10.4)  4.9 (2.3-9.1)  6.2 (3.2-10.6) | 0.5 (0.0-2.6)  0.0 (0.0-2.0)  0.0 (0.0-1.9)  0.0 (0.0-2.3) |

Legend: *Physical, emotional, or sexual. †Prior miscarriage, stillbirth, or termination due to foetal abnormalities. ‡Prior inability to become pregnant after ≥12 months of trying or seeking of medical/professional help for infertility.
