# Supplementary material for: Women’s knowledge, attitudes and views of preconception health and intervention delivery methods: a cross-sectional survey
Source: BMC Pregnancy Childbirth. 2022 Sep 24;22:729. doi: 10.1186/s12884-022-05058-3 (PMC9508727; doi:10.1186/s12884-022-05058-3)
Supplement: Supplementary file 6 — Additional file 6. Women’s attitudes towards preconception health. [file 12884_2022_5058_MOESM6_ESM.docx]

**Additional file 6: Women’s attitudes towards preconception health**

| **Attitudinal variable** | **Response categories** | **N** | **% (95% confidence interval)** |
| --- | --- | --- | --- |
| **Perceived awareness of preconception risk factors** | Not aware at all | 72 | 8.6 (6.9-10.7) |
|  | Slightly aware | 244 | 29.2 (26.2-32.4) |
|  | Moderately aware | 297 | 35.6 (32.4-38.9) |
|  | Very aware | 222 | 26.6 (23.7-29.7) |
|  | *Missing* | *0* |  |
| **Perceived importance of preconception health** | Strongly disagree | 5 | 0.6 (0.2-1.4) |
|  | Disagree | 9 | 1.1 (0.6-2.1) |
|  | Neither agree nor disagree | 47 | 5.6 (4.3-7.4) |
|  | Agree | 412 | 49.3 (46.0-52.7) |
|  | Strongly agree | 362 | 43.4 (40.0-46.7) |
|  | *Missing* | *0* |  |
| **Interest in knowing more about preconception health** | Not at all interested | 165 | 19.9 (17.3-22.7) |
|  | Slightly interested | 208 | 25.0 (22.2-28.1) |
|  | Moderately interested | 267 | 32.1 (29.0-35.4) |
|  | Very interested | 191 | 23.0 (20.2-26.0) |
|  | *Missing* | 4 |  |
| **Preconception health self-efficacy** | Strongly disagree | 3 | 0.4 (0.1-1.1) |
|  | Disagree | 6 | 0.7 (0.3-1.6) |
|  | Neither agree nor disagree | 63 | 7.6 (6.0-9.6) |
|  | Agree | 383 | 46.1 (42.7-49.5) |
|  | Strongly agree | 376 | 45.3 (41.9-48.7) |
|  | *Missing* | 4 |  |
| **Preconception lifestyle change intentions** | Very unlikely | 13 | 1.6 (0.9-2.7) |
|  | Quite unlikely | 43 | 5.2 (3.9-6.9) |
|  | Neither likely nor unlikely | 67 | 8.1 (6.4-10.1) |
|  | Likely | 313 | 37.7 (34.4-41.0) |
|  | Very likely | 395 | 47.5 (44.1-50.1) |
|  | *Missing* | *4* |  |
