# Supplementary material for: Women’s knowledge, attitudes and views of preconception health and intervention delivery methods: a cross-sectional survey
Source: BMC Pregnancy Childbirth. 2022 Sep 24;22:729. doi: 10.1186/s12884-022-05058-3 (PMC9508727; doi:10.1186/s12884-022-05058-3)
Supplement: Supplementary file 7 — Additional file 7. Acceptability and use of intervention delivery methods (full breakdown of participant ratings for each delivery method, in percentages). [file 12884_2022_5058_MOESM7_ESM.docx]

**Additional file 7: Acceptability and use of intervention delivery methods (full breakdown of participant ratings for each delivery method, in percentages)**

1. ***Acceptability of providing preconception health information in various places and settings***

|  | **Very acceptable** | **Somewhat acceptable** | **Neither acceptable nor unacceptable** | **Somewhat unacceptable** | **Very unacceptable** |
| --- | --- | --- | --- | --- | --- |
| Television | 68.0 | 23.9 | 5.8 | 1.3 | 1.0 |
| Billboards & posters | 58.7 | 24.8 | 12.3 | 3.0 | 1.3 |
| Radio | 60.8 | 23.8 | 11.8 | 2.8 | 0.8 |
| Printed material in healthcare settings | 96.0 | 2.5 | 1.0 | 0.2 | 0.2 |
| Social media | 69.2 | 19.0 | 8.0 | 2.2 | 1.7 |
| Personal text or email (e.g. from a GP) | 57.6 | 26.8 | 9.1 | 5.2 | 1.3 |
| Preconception websites/apps | 96.9 | 2.6 | 0.2 | 0.0 | 0.2 |
| With tampons & sanitary pads | 48.8 | 23.8 | 12.7 | 10.6 | 4.1 |
| With pregnancy tests | 77.1 | 13.9 | 5.0 | 2.6 | 1.3 |
| Health education in schools | 82.2 | 12.1 | 3.6 | 1.7 | 0.4 |
| The workplace | 27.4 | 23.7 | 25.9 | 16.8 | 6.1 |

1. ***Acceptability of discussing preconception health with various people***

|  | **Very comfortable** | **Somewhat comfortable** | **Neither comfortable nor uncomfortable** | **Somewhat uncomfortable** | **Very uncomfortable** |
| --- | --- | --- | --- | --- | --- |
| General practitioner | 71.7 | 21.8 | 4.4 | 1.7 | 0.4 |
| Practice nurse | 70.3 | 23.3 | 5.0 | 1.2 | 0.2 |
| Obstetrician/Gynaecologist | 72.9 | 19.1 | 6.4 | 1.1 | 0.5 |
| Midwife | 84.5 | 12.5 | 2.5 | 0.4 | 0.1 |
| Health visitor | 61.7 | 25.0 | 9.4 | 2.9 | 1.0 |
| Pharmacist | 17.9 | 30.9 | 28.7 | 16.7 | 5.9 |
| Dentist | 11.8 | 12.1 | 32.5 | 23.2 | 20.4 |
| Community/family support worker | 27.1 | 24.4 | 32.5 | 11.3 | 4.7 |
| Sexual health/family planning staff | 55.4 | 24.3 | 14.6 | 4.0 | 1.8 |
| Hairdresser/Beautician | 7.6 | 10.6 | 30.8 | 24.5 | 26.6 |
| Friends | 56.6 | 30.0 | 9.5 | 3.5 | 0.5 |
| Family/partner | 84.1 | 12.1 | 2.8 | 0.5 | 0.5 |

1. ***Last contact with various people***

|  | **Within the last week** | **Within the last month** | **Within the last year** | **1-3 years ago** | **4-5 years ago** | **F. More than 5 years ago** | **G. Never** |
| --- | --- | --- | --- | --- | --- | --- | --- |
| General practitioner | 10.9 | 26.5 | 47.1 | 13.0 | 0.8 | 1.1 | 0.6 |
| Practice nurse | 5.2 | 17.4 | 45.7 | 23.6 | 2.1 | 2.7 | 3.3 |
| Obstetrician/Gynaecologist | 1.5 | 2.2 | 12.7 | 17.3 | 5.8 | 12.0 | 48.5 |
| Midwife | 1.7 | 1.7 | 8.0 | 11.9 | 4.9 | 16.3 | 55.6 |
| Health visitor | 1.6 | 1.7 | 9.2 | 10.7 | 3.9 | 15.6 | 57.4 |
| Pharmacist | 7.0 | 18.3 | 41.4 | 18.5 | 3.4 | 2.8 | 8.5 |
| Dentist | 3.2 | 11.0 | 39.7 | 32.2 | 5.1 | 3.2 | 5.7 |
| Community/family support worker | 0.5 | 0.7 | 2.0 | 3.8 | 2.1 | 4.0 | 87.0 |
| Sexual health/family planning staff | 0.4 | 1.0 | 6.6 | 13.1 | 7.4 | 22.4 | 49.2 |
| Hairdresser/Beautician | 5.6 | 14.1 | 50.6 | 16.7 | 1.3 | 2.7 | 9.0 |
| Friends | 88.1 | 7.5 | 2.7 | 0.9 | 0.1 | 0.6 | 0.1 |
| Family/partner | 94.4 | 2.2 | 1.1 | 0.5 | 0.1 | 0.4 | 0.5 |
